# What is the ability of inflamed endothelium to uptake exogenous saturated fatty acids? A proof-of-concept study using spontaneous Raman, SRS and CARS microscopy

**DOI:** 10.1007/s00018-022-04616-4

**Published:** 2022-11-15

**Authors:** Aleksandra Borek-Dorosz, Anna Pieczara, Krzysztof Czamara, Marta Stojak, Ewelina Matuszyk, Katarzyna Majzner, Krzysztof Brzozowski, Arianna Bresci, Dario Polli, Malgorzata Baranska

**Affiliations:** 1 Faculty of Chemistry, Jagiellonian University, 2 Gronostajowa Str., 30-387 Krakow, Poland; 2 Jagiellonian Centre for Experimental Therapeutics (JCET), Jagiellonian University, 14 Bobrzynskiego Str., 30-348 Krakow, Poland; 3 Physics Department, Politecnico di Milano, Piazza Leonardo da Vinci, 32, 20133 Milan, Italy; 4 Institute for Photonics and Nanotechnology at CNR (CNR-IFN), Piazza Leonardo da Vinci, 32, 20133 Milan, Italy

**Keywords:** Endothelium, Lipid droplets, Raman imaging, Fluorescence, Atomic force microscopy, Coherent Raman microscopy

## Abstract

Endothelial cells (EC) in vivo buffer and regulate the transfer of plasma fatty acid (FA) to the underlying tissues. We hypothesize that inflammation could alter the functionality of the EC, i.e., their capacity and uptake of different FA. The aim of this work is to verify the functionality of inflamed cells by analyzing their ability to uptake and accumulate exogenous saturated FA. Control and inflammatory human microvascular endothelial cells stimulated in vitro with two deuterium-labeled saturated FA (D-FA), i.e., palmitic (D_31_-PA) and myristic (D_27_-MA) acids. Cells were measured both by spontaneous and stimulated Raman imaging to extract detailed information about uptaken FA, whereas coherent anti-Stokes Raman scattering and fluorescence imaging showed the global content of FA in cells. Additionally, we employed atomic force microscopy to obtain a morphological image of the cells. The results indicate that the uptake of D-FA in inflamed cells is dependent on their concentration and type. Cells accumulated D-FA when treated with a low concentration, and the effect was more pronounced for D_27_-MA, in normal cells, but even more so, in inflamed cells. In the case of D_31_-PA, a slightly increased uptake was observed for inflamed cells when administered at higher concentration. The results provide a better understanding of the EC inflammation and indicate the impact of the pathological state of the EC on their capacity to buffer fat. All the microscopic methods used showed complementarity in the analysis of FA uptake by EC, but each method recognized this process from a different perspective.

## Introduction

The endothelium is a highly specialized organ composed of a monolayer of cells, lining the blood and lymph vessels [1]. Therefore, it is called a natural gatekeeper between the blood components and the vascular wall [2, 3]. Endothelial cells (EC) are multifunctional and involved in the maintenance of non-thrombogenic properties of the intravascular vessel surface, wound healing, angiogenesis, regulation of vascular tone, cytokines release, leukocyte trafficking, and many others [4].

In recent years, many reports can be found on the important role of EC in physiological and pathophysiological processes [5, 6]. The endothelial dysfunction (ED) can be characterized by an alteration of its action toward pro-thrombotic properties, reduced vasodilation, and pro-inflammatory state [7]. ED is a trigger of various lifestyle diseases such as atherosclerosis [8, 9], diabetes [9, 10], cardiovascular disorders [9], hypertension [11, 12] or Alzheimer’s disease [13, 14]. To understand better the processes that occur in dysfunctional endothelium, in vitro studies are commonly carried out [15–17]. They help to develop strategies to diagnose diseases at the early stage or to slow their development. For example, in coronary artery diseases, the reversal of ED might decrease the risk of acute cardiovascular events [18].

ED can be manifested by inflammation [7, 19, 20]. Many inflammatory agents can be used to invoke the inflammation in the EC, i.e. lipopolysaccharides or cytokines, such as interleukin 6, interleukin 1β, or tumor necrosis factor α (TNF-α) [21–24]. The latter is a transmembrane protein [23] mediating inflammatory effects, which is mainly expressed by activated macrophages [25, 26]. TNF-α may trigger numerous signaling pathways in EC, but the main consequences of its action are increased secretion of intracellular adhesion molecules [27], elasticity decrease [28], increased production of reactive oxygen species [29], and, at higher concentration, the apoptosis [30]. Since dysfunction of EC leads to alterations of their proper functions, it may affect the uptake processes and change of cellular lipidomics.

Fatty acids (FA) are important biomolecules due to their role as a source of energy or substrates for membrane synthesis, but they also have detrimental effects under the condition of FA overload [31]. Omega-3 and omega-6 FA may affect differently the functioning of EC [32]. Omega-6 FA contributes more to inflammatory responses and activation of EC [32], in contrast to omega-3 FA, which may decrease these processes and therefore decrease the risk of cardiovascular diseases [33, 34]. Palmitic acid (PA) is the most abundant saturated FA in the human body [35], however, it can be associated with ED via necroptosis and inflammation [31].

Myristic acid (MA) can be found in human cellular membranes but is present in slightly less amount than PA or stearic acid [36]. MA is also the third most common FA in the human diet [37], and in recent years it has been identified as a predictor of nonalcoholic steatohepatitis. The effect of PA and MA on the human body was investigated [37, 38] and their synergistic effect was suggested. Zock et al. demonstrated the action of these two FA by giving volunteers a special diet. [37] They showed that both MA and PA increased total cholesterol in the human body, but the MA effect on cholesterol-raising was 1.5 times greater than for PA. Martinez et al. also described the influence of these two FA presented in the diet on the human body. Consuming diets enriched with PA and MA causes the development of lipodystrophy and chronic liver disease, macrovesicular steatosis, which may affect hepatomegaly and liver injury. On the other hand, unlike diets high in other FA, diets with MA and PA do not increase body weight but cause inflammation and fibrosis indicative of progressive non-alcoholic fatty liver disease. Moreover, differences in the impact of these two FA were found, MA alone causes an increase in the expression of stress markers in the endothelium [38].

The role of the endothelium in lipid metabolism and its transport is also known, and the concept of the fat buffering by EC has been developed [39]. Despite many studies on the role of lipid droplets (LD) in EC, their biogenesis and function are still not fully elucidated. Various mechanisms of lipid transport (e.g., diffusion or protein-mediated transcellular transport) and LD function in EC formed in vitro and in vivo have been proposed. Previously we have shown that FA uptake results in the formation of esterified LD, thus supporting the hypothesis of the role of LD is the protection of EC from FA-induced endoplasmic reticulum (ER) stress and apoptosis since free FA can be toxic [40–42]. The formation of neutral triacylglycerols minimizes this issue. Kuo et al. showed a correlation between decreased plasma lipids and increased LD in the aortic wall, indicating that LD plays a role in buffering FA in the underlying tissues [47]. LD may be also a source of energy for EC, but it should be noted that ATP production in EC mostly comes from glycolysis and the contribution of lipids is limited.

The uptake of FA may also influence the stiffness or fluidity of cells [43]. To monitor the changes in the mechanical properties of cells, Atomic Force Microscopy (AFM) can be used [44]. Generally, omega-3 FA increases the fluidity of the cell membranes and decreases bending stiffness [45]. As it was mentioned before, the incubation with arachidonic acid causes an increased production of LD in cells and affects the EC stiffness [46].

The application of Raman imaging to study changes in lipid content in vitro is the method of choice due to the large cross-section for Raman scattering and the non-polar nature of these compounds (highly polarizable C–H and C–C bonds). The advantage of Raman microscopy is not only a possibility of visualizing individual cell components at the subcellular level, but also obtaining information about the complex chemical composition of cells. The impact of TNF-α on EC has been previously investigated with the use of Raman spectroscopy [1, 41, 47–50]. The most important feature of the pro-inflammatory properties of TNF-α on EC is the formation of unsaturated LD [1]. As already mentioned, EC incubation with selected FA resulted in cell inflammation [35, 54], which was manifested in the formation of LD [46].

In this paper, we investigate the uptake of two saturated FA by normal and inflamed EC, using a multimodal approach based on Raman, fluorescence, and AFM imaging. Cell analysis is performed first on the microscale followed by nanoscale measurements. We used two deuterium-labeled FA (D-FA), i.e. deuterated palmitic (D_31_-PA) and myristic (D_27_-MA) acids, as sensitive Raman probes to track their accumulation in human microvascular endothelial cells (HMEC-1). TNF-α was used to model inflammation in EC. In contrast to previous studies where EC was incubated with FA, here we examined whether inflamed cells have an altered ability to uptake FA. To make sure we were able to distinguish between endogenous and exogenous FA, we used labeled FA.

## Materials and methods

### Cell culture

HMEC-1 was obtained from the American Type Culture Collection (ATCC, Rockville, Maryland, MD, USA). HMEC-1 were cultured in MCDB131 medium (Gibco Life Technologies) supplemented with 10 mM L-glutamine (Gibco Life Technologies), 1 μg·mL^−1^ hydrocortisone (Sigma Aldrich), 10 mg·mL^−1^ epidermal growth factor (EGF, Sigma Aldrich), 10% fetal bovine serum (FBS, Gibco Life Technologies) and antibiotic antimycotic solution (AAS with 10.000 U penicillin, 10 mg streptomycin and 25 μg amphotericin B per mL). Cells were maintained in an incubator at 5% CO_2_ in air and 37 °C. HMEC-1 cells were regularly tested for Mycoplasma contamination (Lonza, Basel, Switzerland). The cells for Raman measurements were directly seeded onto CaF_2_ slides (25 × 2 mm, Crystran LTD, UK) at a concentration of 2 × 10^5^ cells per well. Cells were kept in a complete MCDB131 medium (Gibco Life Technologies) and left to grow for 24 h. Then, cells were divided into two groups, and one was exposed to 10 ng·mL^−1^ tumor necrosis factor-alpha (TNF-α, Sigma Aldrich) for 24 h, and the second remain untreated. Subsequently, cells of both groups were incubated with 50 µM and 400 µM of D_31_-PA or D_27_-MA for next 24 h. Before supplementation, both FA were saponified with NaOH (20 mM, Sigma Aldrich) and conjugated to 5% FA-free bovine serum albumin (BSA, Sigma Aldrich). HMEC-1 incubated with BSA maintained in the medium was used as a control. After stimulation, cells were washed twice with PBS and fixed with a 2.5% solution of glutaraldehyde (Sigma Aldrich) in PBS for 4 min. The samples were stored in the PBS buffer at 4 °C until Raman measurements.

### Fluorescence imaging

The HMEC-1 were seeded onto 96-well plates at a concentration of 3 × 10^4^ cells per well, in 200 µL of medium per well and left for 24 h to grow, and then cells were treated with D_27_-MA/D_31_-PA at two concentrations, i.e. 50 and 400 µM, for the next 24 h with/without TNF-α pre-incubation. Then, cells were fixed (2.5% glutaraldehyde, 4 min) and directly stained with Phycoerythrin (PE)-labeled mouse anti-human ICAM-1 antibody (BD Pharmingen, San Jose, CA, USA) and BODIPY 493/503 (4,4-Difluoro-1,3,5,7,8-Pentamethyl-4-Bora-3a,4a-Diaza-s-Indacene, Molecular Probes, USA) to visualize ICAM-1 and lipid droplets, respectively, according to the manufacturer protocols, and stained with Hoechst 33,342 (Life Technologies) for 30 min. The expression of surface ICAM-1 molecule (intercellular adhesion molecule 1), LD and cell nuclei were observed by CQ1 Confocal Quantitative Image Cytometer (Yokogawa, Japan) in randomly selected 6 visual fields of view for each well. The images were analyzed using Columbus 2.4.2 Software (Perkin Elmer). Experiments were performed in triplicate and repeated five times.

### Raman imaging

Raman imaging was performed using a WITec Confocal Raman Imaging system (WITec alpha300, Ulm, Germany) supplied with a UHTS 300 spectrograph (600 grooves·mm^−1^ grating, resolution of ca. 3 cm^−1^) and a CCD detector (Andor, DU401A-BV-352). The air-cooled solid-state laser of 532 nm with the application of maximum laser power at the sample position (ca. 30 mW) was used together with the 63 × water immersion objective (Zeiss Fluor, NA = 1.0). For each cell, a 0.5-s exposure time per spectrum was applied and a sampling density of 0.5 μm in the x/y plane. Imaged areas of at least 7 cells per group in two independent experiments for each group were measured.

Raman spectra of lipid standards were obtained with the application of the 20 × air objective (Zeiss EC Epiplan, NA = 0.45) after placement of a pinch of standard sample on CaF_2_ slide. Each spectrum was excited with a maximum laser power at sample position collecting 10 scans using an integration time of 0.5 s.

### CARS imaging

A home-built nonlinear optical microscope was used for CARS imaging [51, 52]. It features a mode-locked Erbium:fiber-based oscillator (FemtoFiberPro, TOPTICA Photonics AG, Germany) working at 40-MHz repetition rate. The multi-branch laser source delivers 1560 nm pulses through which pump and Stokes pulses for coherent Raman imaging are generated. As for the pump, one of the beams is frequency doubled via a 10-mm-long MgO-doped periodically poled lithium niobate (PPLN) crystal with a poling period of 19.3 μm to generate 778 nm pulses with 1-ps duration. Another laser branch is employed to generate 1-ps Stokes pulses, tunable in the 950–1050 nm range: a highly nonlinear fiber (HNLF) broadens the laser spectrum, then frequency-doubling and wavelength selection occur by spanning the poling period of a fan-out 10-mm-long PPLN crystal (i.e., 26–33 μm) via a motorized stage.

The spatiotemporally superimposed beams are sent to an inverted configuration microscopy unit. A 100x 1.25-NA water-immersion objective (C-Apochromat 100x/1.25 W Corr M27, Zeiss, Germany) illuminates the sample, while a 40× 1.3-NA oil-immersion objective (CFI Super Fluor 40× Oil, Nikon, Japan) collects the CARS signal, measured by a photomultiplier tube (Hamamatsu Photonics, Japan). Imaging occurs by sample scanning through an x–y motorized sample stage, with the specimen placed in between two 0.17-mm-thick quartz slides. Laser powers at the focal plane are 0.5 mW for the Stokes and 7.5 mW for the pump. The pixel dwell time is 1.5 ms. The pixel size is 350 nm. Between 4 and 7 images were recorded for each cell group at different positions of the sample (total number of cells: 30 for control, 32 for TNF-α, 33 for D_31_-PA 50 µM, 47 for TNF-α + D_31_-PA 50 µM, 15 for D_31_-PA 400 µM and 13 for TNF-α + D_31_-PA 400 µM).

### SRS imaging

SRS imaging was performed using the homemade setup located at the Faculty of Chemistry, Jagiellonian University in Krakow. We use a 2-ps laser (Fluence Lazurite) with 20 MHz’s repetition rate based on a fiber laser producing 1029-nm Stokes beam with 450 mW of average power with a built-in optical parametric oscillator (OPO) that produces tunable wavelength beam in the range of 750–950 nm with an average power of 100 mW. The signal is retrieved with the use of *Stanford Research Systems SR865A* lock-in amplifier, sent to the electronics, and stored by the software provided by WITec. In our setup, the Stokes beam is modulated with an acousto-optic modulator (AOM) and the stimulated Raman loss (SRL) signal is collected. An *UPlanFLN* NA 0.75 40× *Olympus* air objective lens was used to focus the laser beams on the sample. The transmitted pump beam was collected by a *MRD07620* 50× NA 1 *Nikon* water-dipping objective lens. The power before the sample was 20 mW. The size of the images was up to 40 $\times$ 40 µm^2^ with a pixel size of 0.33 µm. Pixel dwell time was set to 30 ms.

### AFM imaging

AFM imaging was performed using the Confocal Raman Imaging system (WITec alpha300, Ulm, Germany). AFM was used to investigate topographic and structural changes of EC surface induced by D_27_-MA/D_31_-PA stimulations in pretreated cells with TNF-α. AFM measurements were performed in the tapping (AC) mode with the force modulation probes (*k* = 2.8 N/m, WITec, Ulm, Germany) using the 20× immersive objective (Olympus, NA = 0.5). The AFM resolution was 256 × 256 pixels. AFM data analysis was performed using Gwyddion 2.55. AFM images were corrected using Mean plane subtraction and Median method, based on finding the median of scan lines and showing as representative image height.

### Data analysis and processing

The spectra were pre-processed using a cosmic ray removal (filter size 3 and dynamic factor 8) and baseline subtracted (offset correction) evaluated with a WITec Project PLUS 5.1 software. Then the *k*-means cluster analysis (KMCA) was performed enabling us to group spectra into two clusters that represent nuclei and cytoplasm including all cellular organelles. Additionally, for one representative cell, a detailed analysis was performed, and four independent clusters were separated for nuclei, LD, endoplasmic reticulum area, and cytoplasm. The recorded Raman spectra matrices enabled the visualization of the Raman images of lipids and LD distribution based on the integral intensity of chosen marker bands for lipids, i.e. 2830–2900 cm^−1^ and 2060–2240 cm^−1^. The obtained images were normalized. The average spectra of the whole cytoplasm (protein-rich area) obtained in KMCA were extracted and normalized in the 400–3100 cm^−1^ spectral range. The integral intensities of C–D (2060–2240 cm^−1^) and C–H stretching region for lipids (2830–2900 cm^−1^) were obtained with the D method implemented in OPUS 7.0 software (Bruker).

Statistical significance of all identified changes was tested by the analysis of variance performed in the Origin Pro 9.1 (Origin Lab Corporation) software (ANOVA model with post-hoc Tukey’s test) to quantify the differences in all pairwise comparisons for each group.

CARS images were processed with ImageJ software with built-in functions. All images were equally subjected to background subtraction and duplicated. Next, with the use of intensity thresholding, the images were controlled until only the cytoplasm region with high content of lipids remained visible thanks to the higher resonant signal. The threshold was determined manually in the ImageJ software and kept similar for each analyzed image. The intensity of the areas occupied by lipid components was measured with the “Analyse Particle” function. In the same way, the images were processed to obtain the area occupied by the cells. The threshold was manually specified in ImageJ and kept at a similar level for each image analyzed. The intensity of the areas occupied by lipid components was measured by analysing the function of particles. SRS images were analyzed using a WITec Project PLUS 5.1 software.

## Results and discussion

**Fig. 1:**
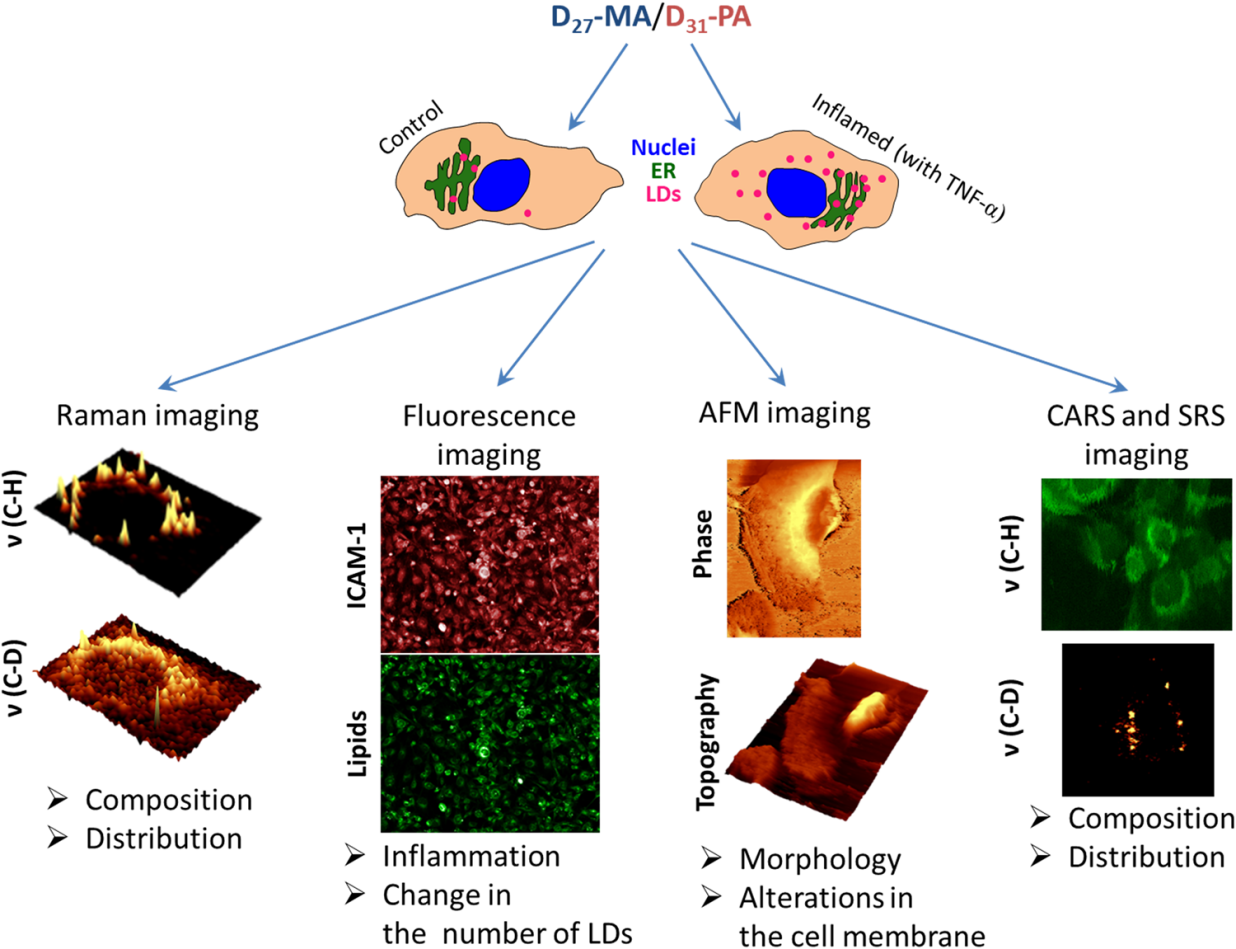
The scheme of experiments and their purpose

The uptake of D_31_-PA and D_27_-MA by HMEC-1 cells was investigated first by Raman imaging, a method that uses the unique spectral signature of the C–D stretching vibration (Fig. 1). Accumulation of D-FA was analyzed in TNF-α pretreated cells in comparison to the (untreated) control. HMEC-1 inflammation was confirmed by immunochemical staining. The effect of D-FA uptake on HMEC-1 cell morphology was further investigated by AFM and two coherent Raman microscopies (SRS and CARS).

### The uptake of fatty acids by endothelial cells depends on the type and concentration of acid, and the condition of the cells

**Fig. 2:**
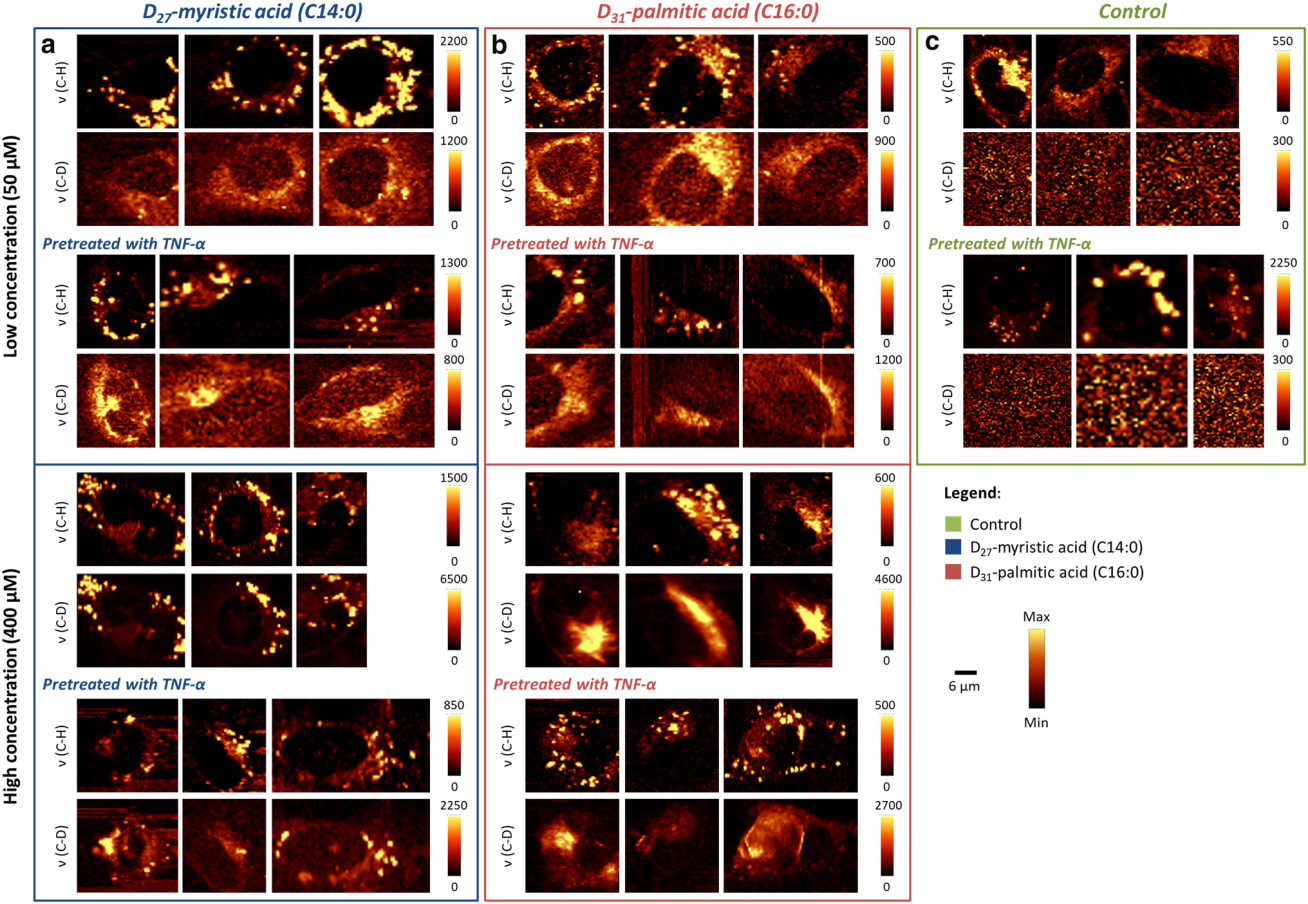
Raman images of HMEC-1 cells upon D-FA uptake in normal and pathological conditions (TNF-α treated cells) HMEC-1 incubated with D_27_-MA (**a**) and D_31_-PA (**b**) at 50 µM and 400 µM concentrations in the reference to control (**c**)

An uptake of D_27_-MA or D_31_-PA by normal and TNF-α pretreated cells was studied at 50 µM and 400 µM concentrations. Raman images provided information about the subcellular localization of endogenous FA and exogenous D-FA, which can be distinguished due to deuterium labeling. Figure 2 shows representative Raman images based on the integration of the *v*(C–H) band in the spectral range 2830–2900 cm^−1^, displaying regions rich in endogenous lipids (i.e., LD and ER), while the spectral range of 2060–2240 cm^−1^ (*v*(C–D)) was used for visualization of spots rich in D-FA. This discrimination is feasible because the C–D stretching vibration occurs in the “silent” spectral region. For the determination of the endogenous lipids and D-FA content, the Raman images based on *v*(C–H) and *v*(C–D) integral intensities were normalized. The scale bars displayed next to the Raman images indicate the maximum intensity of *v*(C–H) or *v*(C–D) Raman bands for all images in a row. This protocol was used to compare data obtained in the same conditions for D_27_-MA and D_31_-PA (presented in the same row), so the intensities indicated on the respective scale bar for the C–H and C–D image for the same experiment (in the same column) are not directly interpreted.

HMEC-1 exhibits the ability to form LD spontaneously in normal conditions, but after treatment with the TNF-α, unsaturated LD are newly formed [1]. This effect was also observed here for all investigated cells, i.e., LD are clearly visible in the Raman images of HMEC-1 based on the intensity of *v*(C–H) vibration (Fig. 2a, b and c).

**Fig. 3:**
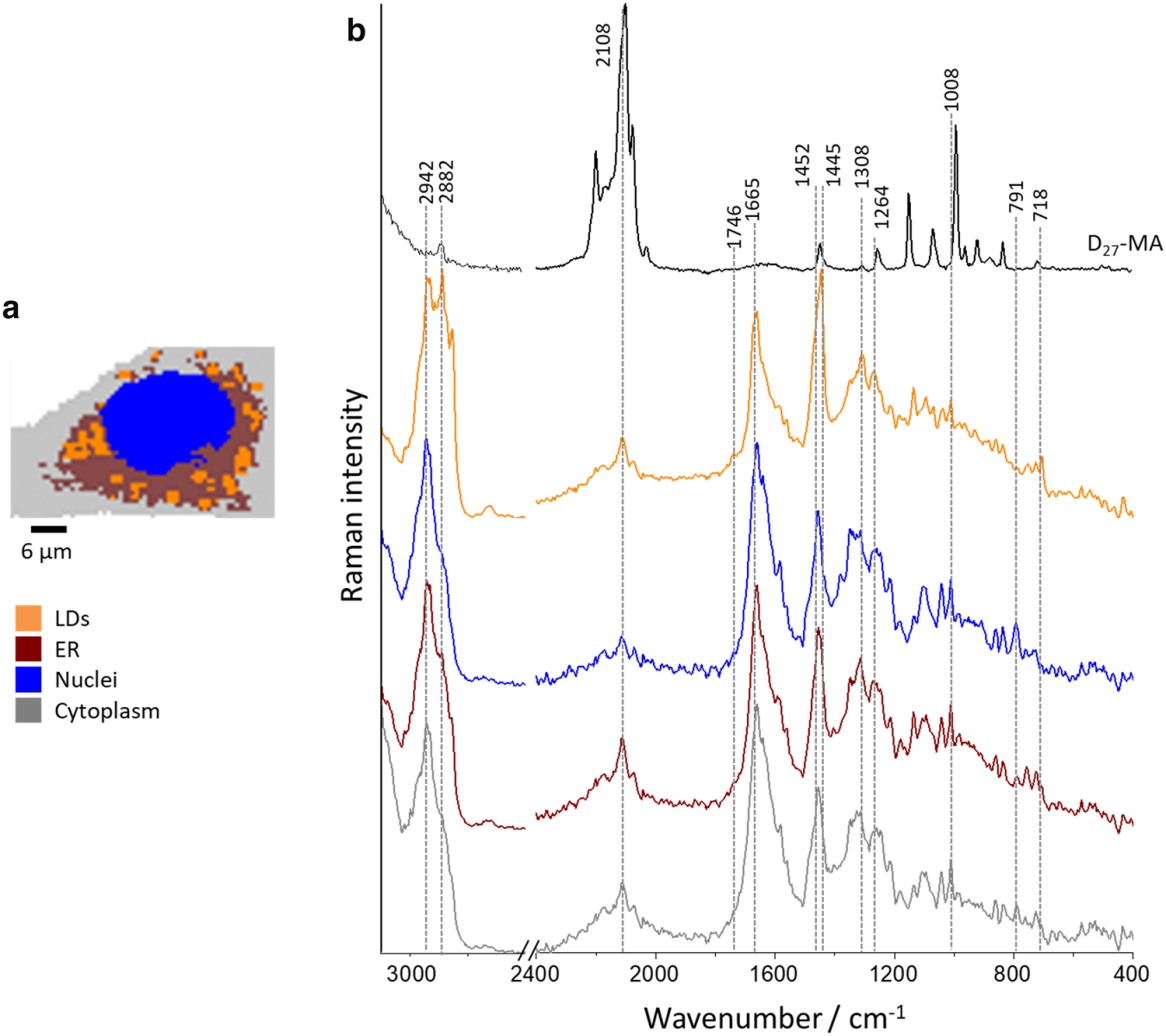
KMCA of representative HMEC-1 incubated with D_27_-MA. The image shows four classes (blue–nucleus, brown–ER-rich area, orange–LD, gray–cytoplasm) (**a**). The average Raman spectra of the respective classes and spectrum of D_27_-MA (black, **b**). Scale bar equals 6 µm. All spectra were maximally extended on the y-axis

After incubation with D-FA at low concentration (50 µM), the *v*(C–D) signal can be seen widely spread in the cytoplasm (Fig. 2a and b), mostly in the area of ER (vide supra Fig. 3), with only a few evidently formed LD. Incubation with a higher concentration of D-FA (400 µM) results in the *v*(C–D) signal being omnipresent in the cytoplasm for D_31_-PA, while for D_27_-MA-treated cells LD are evidently visible. Additionally, looking at the intensity of the *v*(C–D) signal in cells incubated with D_27_-MA at a lower concentration, it can be noticed that their TNF-α stimulation led to increased D-FA uptake compared to unstimulated cells. On the other hand, when comparing the action of this acid in higher concentrations, the situation seems to be opposite. For D_31_-PA, it is difficult to interpret images collected by spontaneous Raman spectroscopy to clearly define the difference between stimulated cells in normal and pathological conditions, for both low and high concentrations. The number of cells measured was too small to make clear conclusions.

Raman images may be ambiguous and difficult to interpret when the assessment is based on visual inspection, so chemometric analysis of the lipidic part of cells was performed. To obtain a spectral profile of individual subcellular compartments, Raman data were subjected to KMCA. Representative KMCA of HMEC-1 incubated with D_27_-MA is shown in Fig. 3. It was possible to distinguish four cellular compartments, i.e., the nucleus (blue class, based on 785 cm^−1^ DNA/RNA marker band), the ER area (brown class, based on a high contribution of lipids, including phospholipids), LD (orange class manifested by high intensity of Raman features at 2852 and 2882 cm^−1^) and the cytoplasm (gray class, with a stronger contribution of proteins in comparison to other subcellular regions). For each class, the average Raman spectrum is shown (Fig. 3b). The characteristic signal originating from *v*(C-D) vibration at 2108 cm^−1^ is clearly seen in the spectrum of each class, with the highest intensity for the ER and the lowest for the nucleus. In the latter case, probably the signal from the cytoplasm above the nucleus contributed here.

The spectrum of LD (orange line, Fig. 3b) contains bands that are seen in the reference spectrum of D_27_-MA, but also other bands due to non-deuterated lipids (1666 cm^−1^ and more), as well as a small intensity shoulder at 1746 cm^−1^ can be noticed and assigned to the esterified form of lipids. From that observation we can hypothesize a passive absorption of free FA by cells (without the participation of transporter proteins, such as albumin) resulting in synthesis and accumulation of triacylglycerols in the cytoplasm of cells. It should be noticed also that all LD show a similar composition, so newly formed LD are composed not only of incorporated D-FA but also of cellular lipids, i.e. phospholipids.

**Fig. 4:**
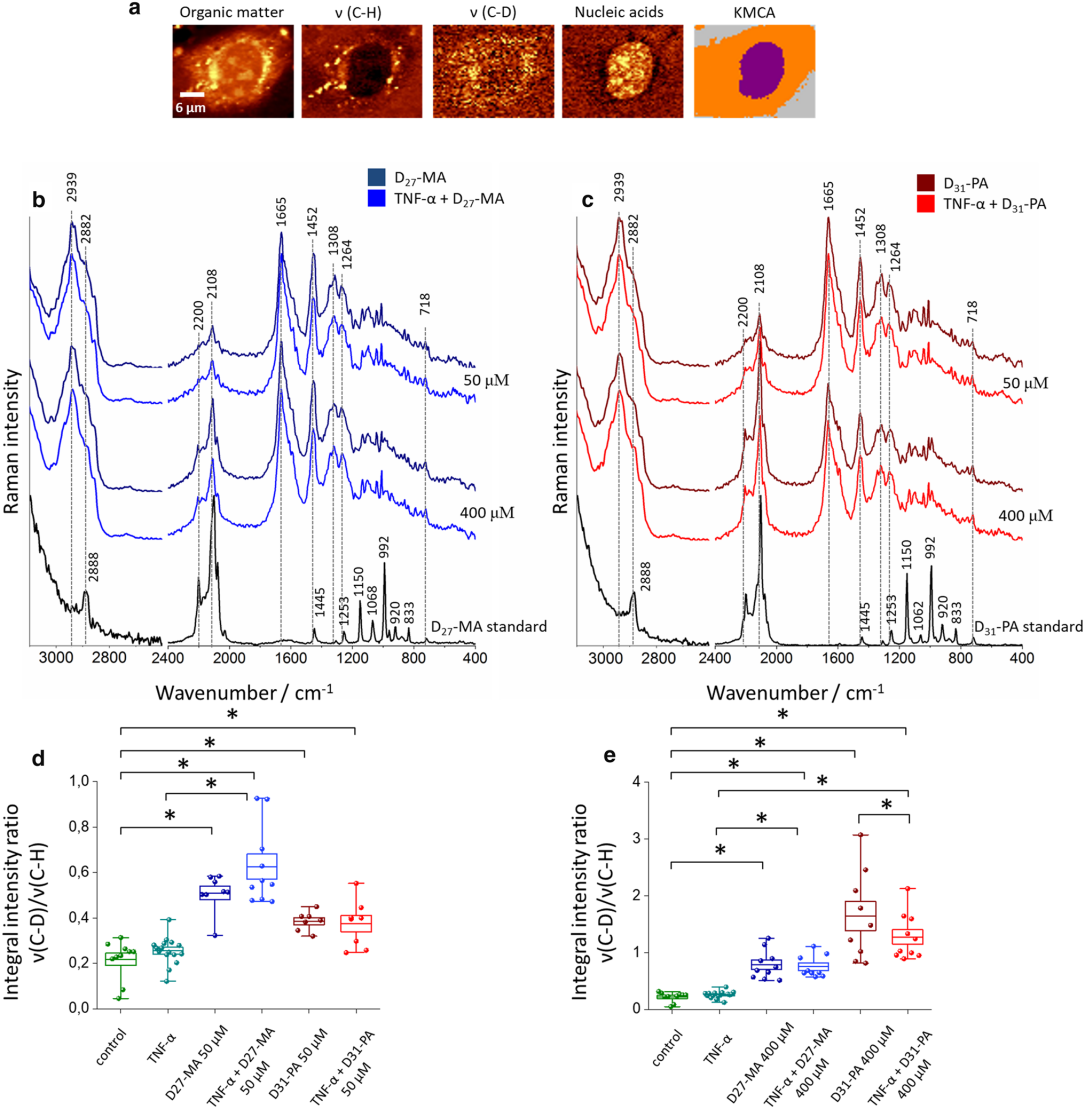
Quantification of the uptake of FA by inflamed HMEC-1 cells. Raman images obtained by integration of bands in the regions: 3030–2830 cm^−1^ (organic matter), 2880–2830 cm^−1^ (v(C–H)), 2150–2050 cm^−1^ (v(C–D)) and 800–770 cm.^−1^ (nucleic acids), together with KMCA image (blue class–nucleus, orange–cytoplasm) (**a**). The average Raman spectra of the cytoplasm of HMEC-1 cells treated with D_27_-MA (**b**) and D_31_-PA (**c**), respectively. Calculated integral intensity ratio for bands originating from v(C–D) and v(C–H) vibrations for low (**d**) and high concentration (**e**) of used FA. The spectra of reference D-FA are also shown. All spectra were maximally extended in the y-axis. *p < 0.05

To compare quantitatively the uptake of D-FA by inflamed cells in reference to control, the Raman spectra taken from cells treated with D-FA were grouped into two classes, i.e., the nucleus and the cytoplasm class, the latter includes ER region and LD together with other organelles (Fig. 4a). This approach enabled us to analyze the overall signal from D-FA originating from the *v*(C–D) vibration since not always LD were distinct enough and lipid signal was rather dispersed in the whole cytoplasm. For each experimental group, an average spectrum is presented in Fig. 4a and b together with the reference spectra of D_27_-MA and D_31_-PA. HMEC-1 treated with a low concentration of D-FA (50 µM) show only slight changes in the intensity of the *v*(C–D) band, i.e., a small increase in the case of cells incubated with D_31_-PA. For cells treated with a high concentration of D-FA (400 µM), for both D_27_-MA and D_31_-PA, the intensity of *v*(C–D) band is lower in the spectra from cells pretreated with TNF-α in comparison to control. To quantify the uptake of D-FA, the ratio of integral intensities of Raman bands originating from *v*(C–H) and *v*(C–D) was calculated (Fig. 4d and e). By doing so, the other effects which could influence the background of the spectra and hence bands intensity was eliminated. For the low concentration of D-FA (Fig. 4d), the uptake of D_27_-MA was greater than D_31_-PA. Such difference in the uptake of those two D-FA may result from the differences in their aliphatic chain length [53]. R.W. Mitchell et al*.* [53] classified D_27_-MA to medium-length saturated FA, while D_31_-PA for a long chain one. They have also shown that the uptake of FA through endothelium monolayer is easier for medium-length FA. Pretreatment with TNF-α enhanced the uptake of D_27_-MA used in low concentration, while did not affect the uptake of D_31_-PA.

For HMEC-1 cells incubated with D-FA in high concentrations, the observations are different. Treatment of EC with a concentration of 400 µM D_27_-MA resulted in its uptake; however, this effect was more pronounced for D_31_-PA. In turn, a decrease of the *v*(C–D) band intensity observed in the spectra collected from cells pretreated with TNF-α and incubated with D_31_-PA may suggest a slight downregulation of the D-FA uptake that occurs upon EC inflammation. The pretreatment with TNF-α did not influence the uptake of D_27_-MA applied in high concentrations.

**Fig. 5:**
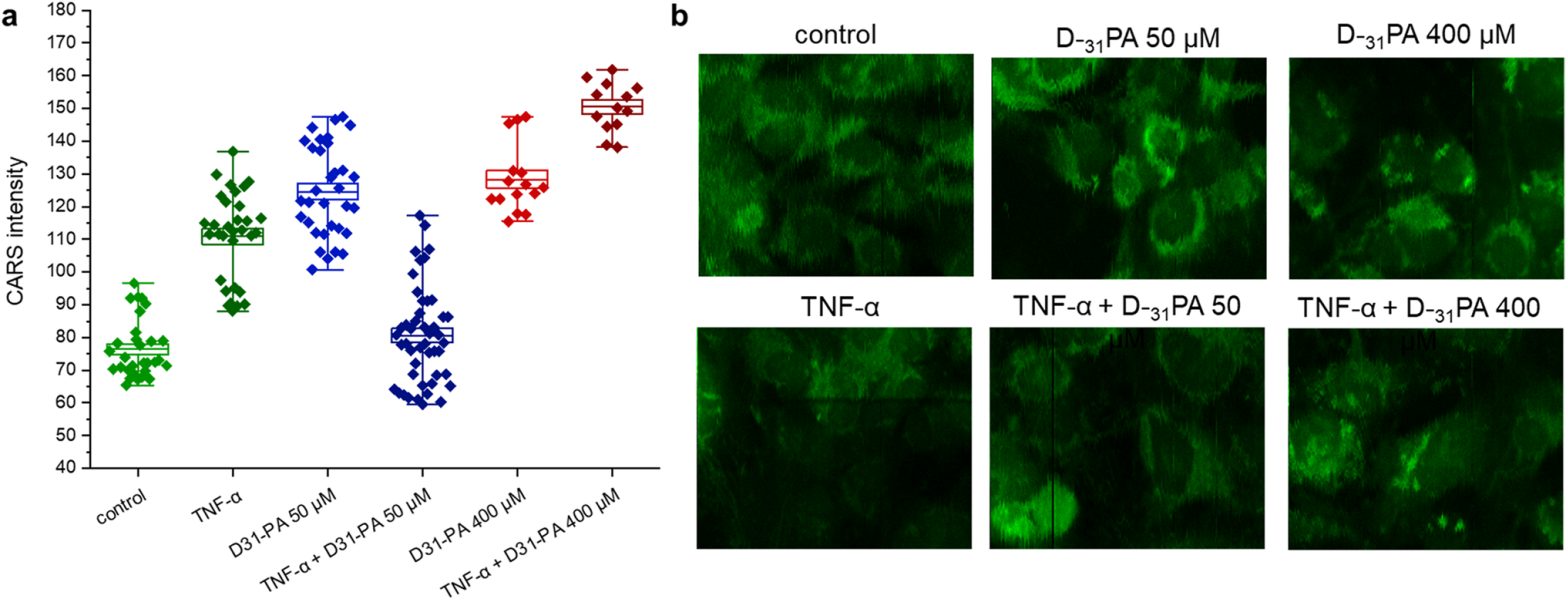
Analysis of CARS images of lipid content in HMEC-1 incubated with D_31_-PA at 50 µM and 400 µM. The quantification of CARS signal at 2850 cm^−1^ (**a**). and representative CARS images (**b**) of HMEC-1 cells upon D_31_-PA uptake in normal and pathological conditions (TNF-α treated cells). Values given as mean ± SEM are shown in box plots: mean (horizontal line), SEM (box), minimal and maximal values (whiskers). *p < 0.05

Since the number of cells measured by Raman microscopy was relatively small to draw definite conclusions, coherent anti-Stokes Raman scattering (CARS) was used, which allowed the measurement of a large population of cells in a much shorter time (Fig. 5). It can be seen that the uptake of D_31_-PA depends on its concentration and the condition of the cells. Inflamed cells show increased D_31_-PA uptake at higher concentrations (400 µM), but not when its concentration is low (50 µM). Here we applied the CARS microscopy that provides information on the overall content of lipids in cells, however, obtained in a label-free manner. Figure 5 presents the quantification of CARS signal at 2850 cm^−1^ intensity for experimental conditions as follows: control (untreated cells), TNF-α treated, D_31_-PA used in concentrations of 50 µM and 400 µM, without and after pretreatment with TNF-α. The CARS signal based on C-H lipid stretching vibrations shows the highest content of lipids in cells treated with TNF-α followed by the addition of a high concentration of D_31_-PA. In turn, the HMEC-1 cells treated with an inflammatory agent followed by the addition of D_31_-PA in low concentration show the content of lipids at the level of control (untreated cells). We anticipated that CARS imaging will provide similar results as extracted from fluorescence images since in both cases the overall lipid content was analyzed. On the other hand, the CARS intensity comes from the intrinsic vibrational modes of lipids (specifically C-H vibration), not from the introduced externally fluorescent label (BODIPY).

**Fig. 6:**
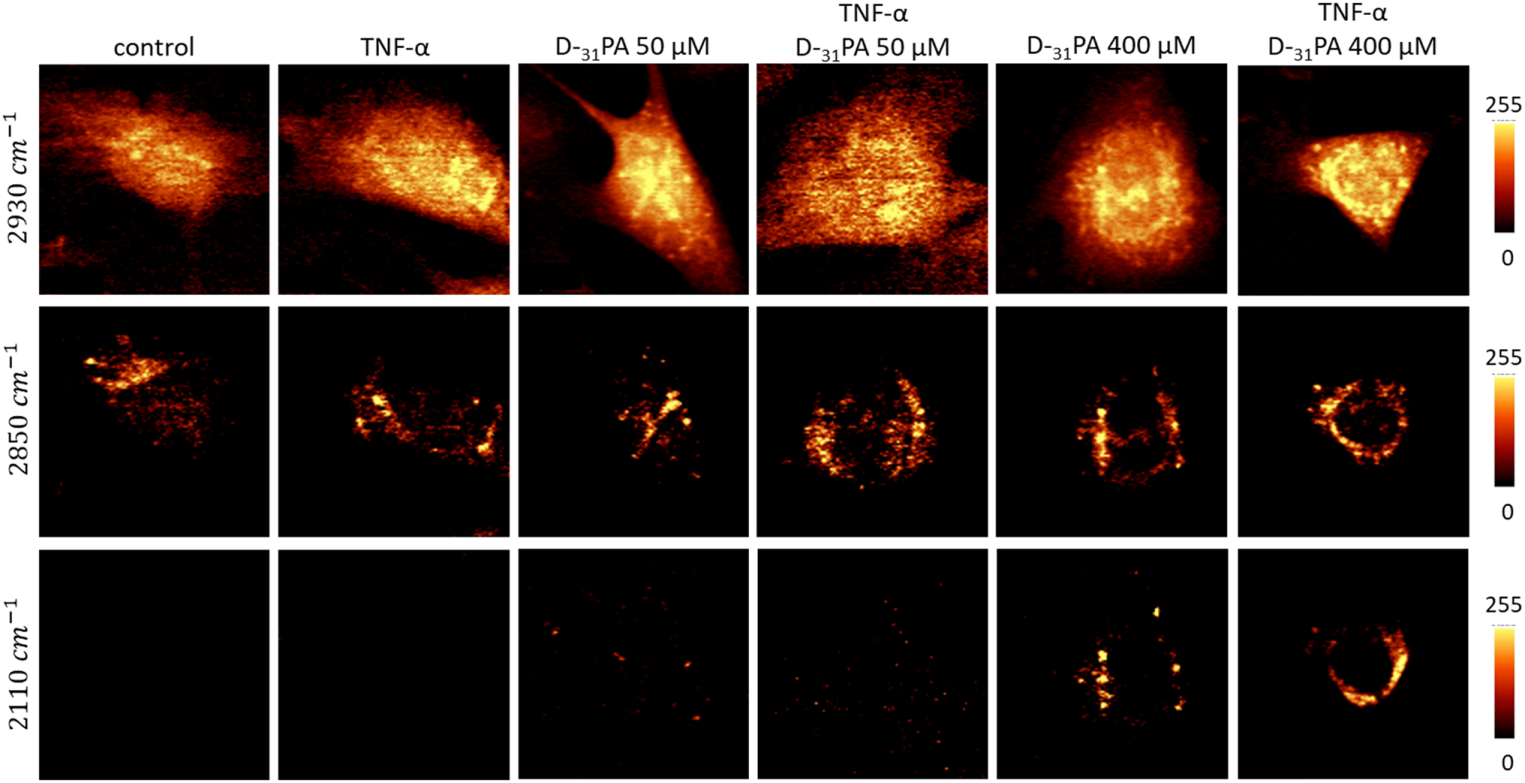
Analysis of SRS images of lipid content in HMEC-1 incubated with D_31_-PA at 50 µM and 400 µM. Representative SRS images of HMEC-1 cells upon D_31_-PA uptake in normal and pathological conditions (TNF-α treated cells)

An uptake of D_31_-PA by untreated and TNF-α pretreated cells was also studied employing SRS (Fig. 6) at 50 µM and 400 µM concentrations, incubated for 24 h. SRS images provided information about the subcellular localization of endogenous FA and exogenous D-PA, which can be distinguished due to deuterium labeling. SRS microscopy allowed imaging lipid and protein and lipid distributions (2930 cm^−1^), mainly lipids (2850 cm^−1^), and solely incorporated D_31_-PA (2110 cm^−1^). As shown in Fig. 6, there is a very large difference in the lipid content in cells depending on the concentration of D_31_-PA employed. The uptake of D_31_-PA is much higher at the higher concentration (400 µM), and this agrees with the Raman and CARS data. At lower concentration of D_31_-PA, both for control and inflamed cells, the signal from D_31_-PA is not clearly visible. Pre-stimulation of EC with TNF-α enhanced the uptake of D_31_-PA used at the higher concentration, while it did not affect the uptake of D_31_-PA at the lower concentration. It is worth mentioning, that the CARS and SRS techniques differ slightly in terms of the interaction of molecular dipoles with the light pulses phenomena and electron transitions. In the CARS process, the sample interacts consecutively with the electric fields of the pump, Stokes and probe (often replaced by a replica of the pump field itself), there is the interaction of 3 photons, and the generation of a new photon of the anti-Stokes frequency (*ω*_as_ = 2*ω*_p_ – *ω*_s_) takes place. On the other hand, in the SRS, the measured signal is a slight modification of the beam intensity (SRG on the Stokes beam or SRL on the pump beam). CARS microscopy was used to study the intercellular distribution of lipids in a large group of cells as this technique is well suited for selective imaging of lipids.

### Uptake of saturated fatty acids decreases the inflammation in endothelial cells

**Fig. 7:**
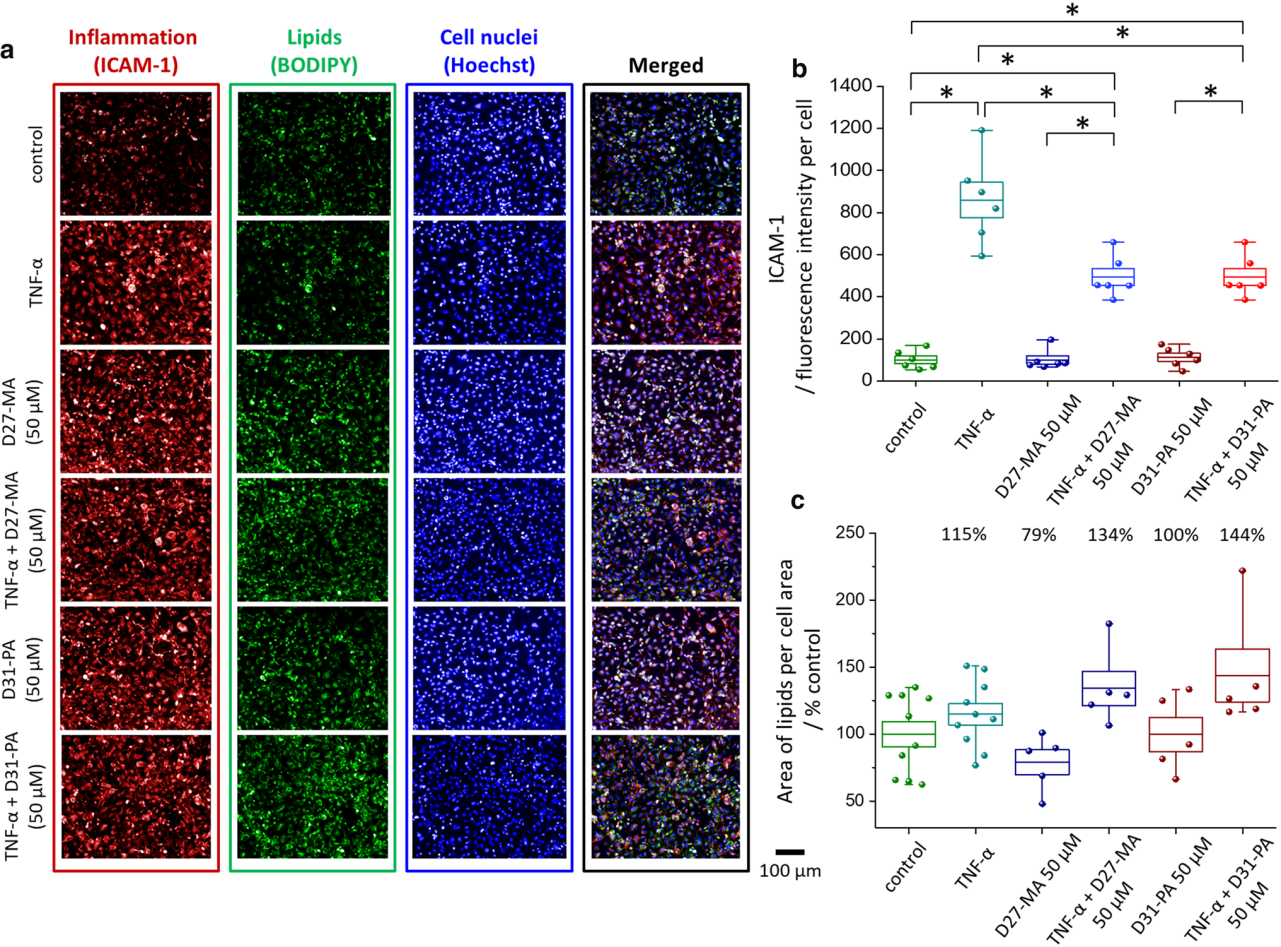
Analysis of inflammation and lipids contribution in HMEC-1 cells incubated with a low concentration of D-FA (50 µM). Representative fluorescence images of HMEC-1 cells treated with D-FA (**a**) showed surface expression of ICAM-1 (red areas), lipids distribution (green areas), and the number of nuclei (blue areas) together with calculated average fluorescence intensity of ICAM-1 (**b**) and area covered by lipids presented as a % of control cells (**c**). Values given as mean ± SEM are shown in box plots: mean (horizontal line), SEM (box), minimal and maximal values (whiskers). *p < 0.05

**Fig. 8:**
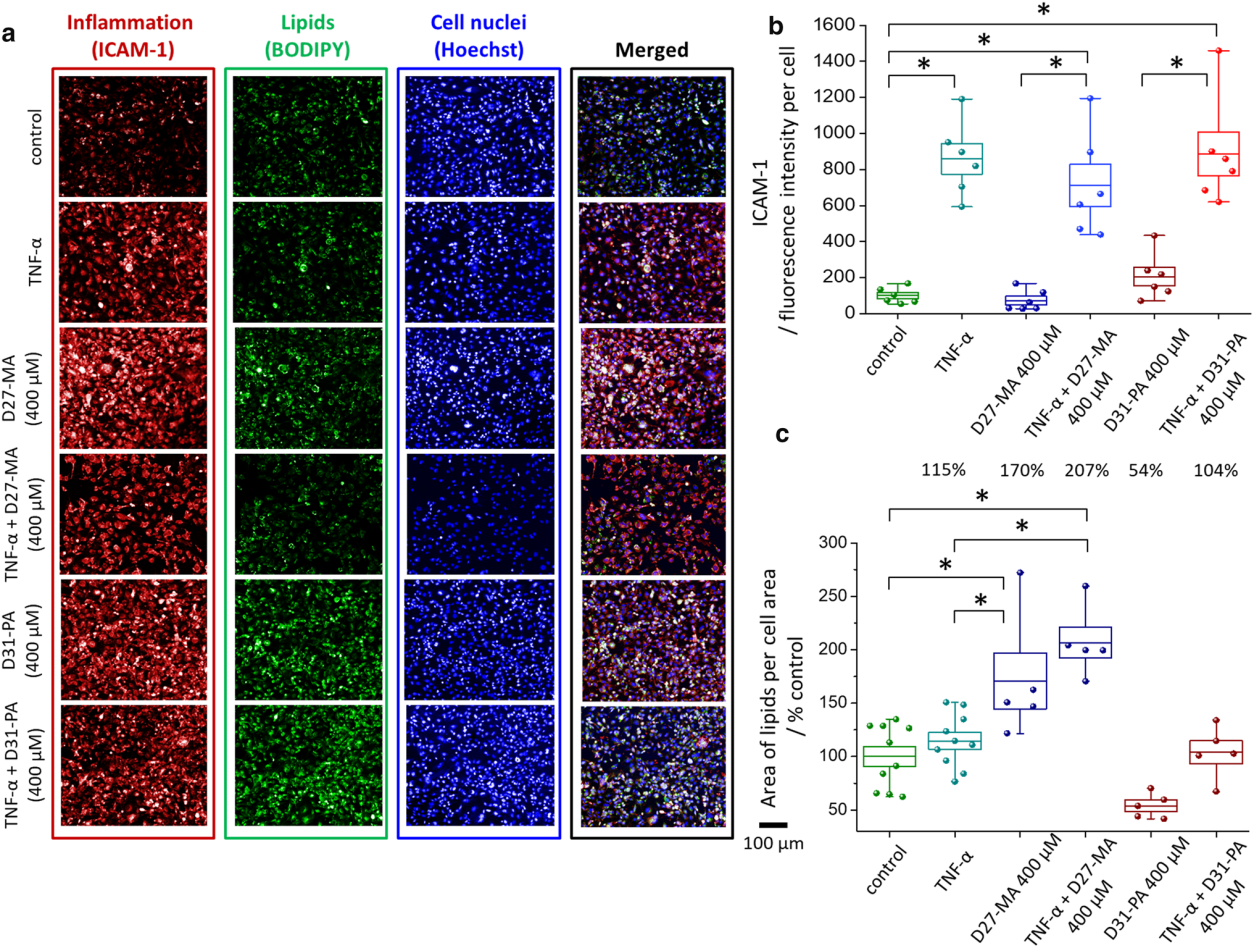
Analysis of inflammation and lipids content in HMEC-1 incubated with D-FA at 400 µM. Representative fluorescence images of HMEC-1 cells (**a**) showed surface expression of ICAM-1 (red areas), lipids (green areas), and nuclei (blue areas) together with calculated statistics of ICAM-1 fluorescence intensity (**b**) and area covered by lipids presented as a % of control cells (**c**). Values given as mean ± SEM are shown in box plots: mean (horizontal line), SEM (box), minimal and maximal values (whiskers). *p < 0.05

To verify whether D-FA stimulate ICAM-1 expression in HMEC-1 cells, fluorescence microscopy was applied. All experimental groups of HMEC-1 treated with D_27_-MA or D_31_-PA for 24 h, for control cells and the one preincubated with TNF-α, were tested on the ICAM-1 expression and lipid content (BODIPY staining). The representative fluorescence images are presented in Figs. 7a and 8a, followed by quantitative analysis of the ICAM-1 surface expression (Figs. 7b and 8b), as well as the analysis of the area covered by lipids (Figs. 7c and 8c) for cells treated with both D-FA at low and high concentrations, respectively.

The analysis of ICAM-1 expression via fluorescence microscopy (Figs. 7a, b and 8a, b) showed that TNF-α evoked the inflammation of EC [21]. Inflammation caused by TNF-α is mediated by the TNFR1 receptor followed by a series of changes in the cell leading to the release of the nuclear factor (NF-kβ) and AP-1 responsible for the initiation of the inflammatory response [54]. The influence of FA on the endothelium is a complex process and is still under investigation. PA is generally considered as lipotoxic FA, which can contribute to inflammation due to induction of interleukin IL-6 [55] and in certain concentrations may lead to cell autophagy [54]. On the other hand, W. Li et al*.* [56] have shown that TNF-α stimulates PA transcytosis across the endothelial barrier. MA, on the other hand, is one of the FA known for the acylation of cellular proteins and does not inhibit cell growth or cause cytotoxicity [57].

In our study, D_27_-MA and D_31_-PA, independently from used concentration, did not cause inflammation of EC (Figs. 7b and 8b). In the pairwise comparison (D-FA vs. D−FA + TNF-α) the effect of inflammation was clearly seen, however inflammation induced by TNF-α did not seem to be enhanced by D-FA.

The changes in lipid content in EC due to the uptake of D-FA were investigated based on BODIPY staining (Figs. 7a and 8a) and calculations of the cell area covered by lipids (Figs. 7c and 8c). EC incubated with TNF-α show a higher content of lipids in a form of newly synthesized LD, as previously reported [1]. In each pair, D-FA vs. D−FA + TNF-α, some differences between both D-FA in a concentration-dependent manner can be noticed. For a low concentration of both D-FA, a similar area of cells was covered by lipids. The cell area covered by lipids calculated for EC treated with 400 µM concentration of D_27_-MA is higher when compared to cells treated with D_31_-PA (Fig. 8c). The results obtained for all samples pretreated with TNF-α showed a higher percentage of cell area covered by lipids. This is most likely caused by the additive effect of TNF-α and D-FA on LD formation. In general, fluorescence microscopy showed that TNF-α is responsible for both the inflammation and in great part for the increase in lipid content in EC. However, using BODIPY staining, no information about D-FA uptake alone was observed. Using fluorescence, the information is given from all lipids in general, both those that are normally present in the cell and those up-taken by cells due to stimulation, while in Raman spectroscopy we infer specifically about the uptake of D-FA.

### Myristic and palmitic acids change differently the morphology of the cellular membrane of endothelial cells

**Fig. 9:**
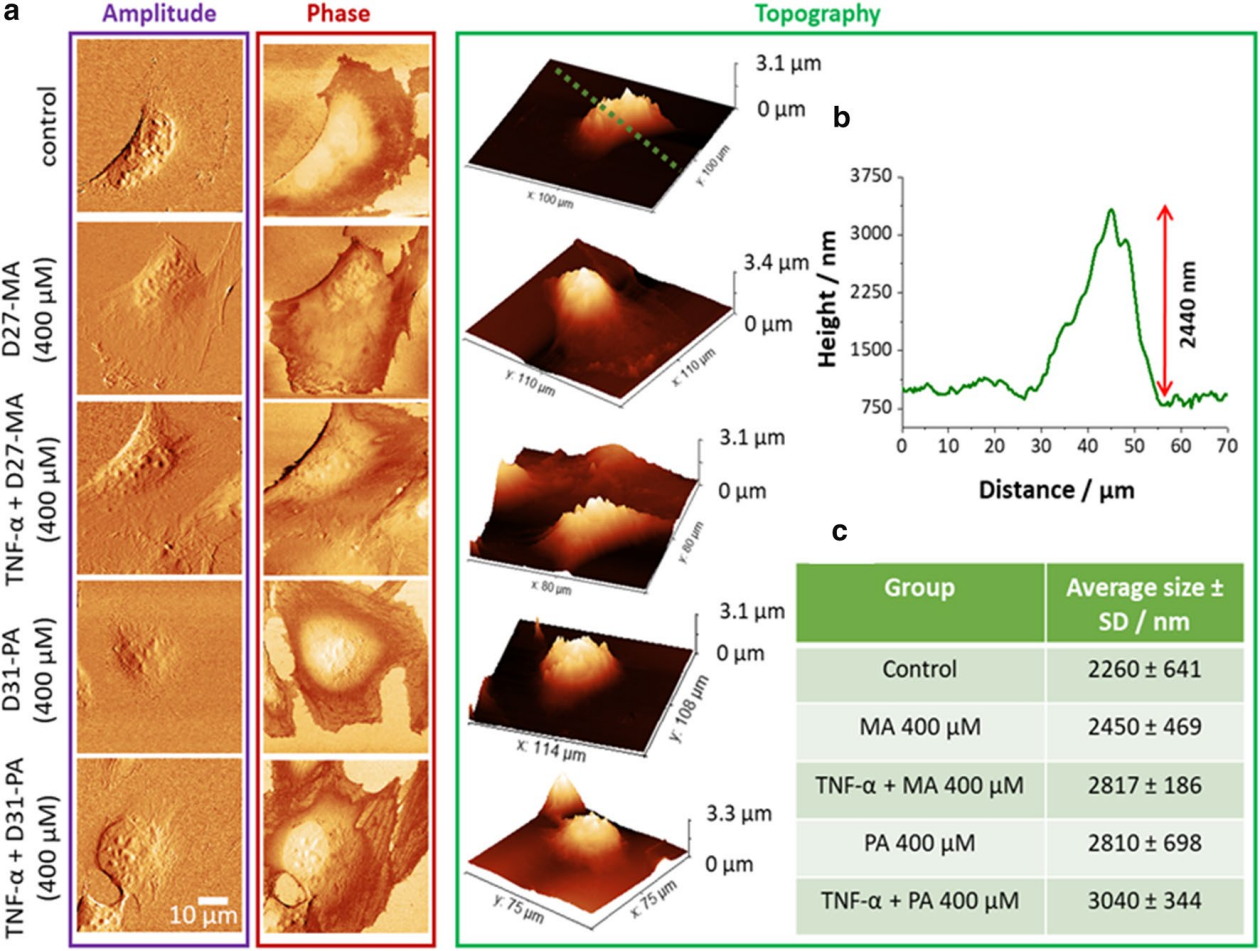
AFM images of HMEC-1 (with and without stimulation with TNF-α) incubated with D-FA at 400 µM for 24 h. Representative images of cells topography, phase, and amplitude (**a**) The height profile (marked in green line) enabled to define changes in the morphology of cells as shown for example (**b**) together with the cell size statistics (**c**)

Atomic force microscopy (AFM) imaging was used to investigate the morphology of the cells after D-FA uptake in the state of their inflammation and control conditions. Images of the amplitude, phase and topography of representative cells incubated with D-FA after TNF-α stimulation as well as without that treatment are shown in Fig. 9.

EC in a normal state and those incubated with D_27_-MA show a characteristic elongated shape, while the cells treated with D_31_-PA are more oval and the entire cell stretches out on the substrate. The differences also manifest themselves in the phase images. D_31_-PA treated cells have a more rugged cell membrane than those from other groups. PA modulates the estrogen receptor alpha variant (ER46), which is located in the plasma membrane, through the nitric oxide synthase (eNOS) pathway in endothelial cells [58, 59] and PA may also lead to cell apoptosis [60]. These two facts may be involved in the changes of the cell shape. The analysis of cell size (Fig. 9b, c) indicates an increase in height of EC treated with D_31_-PA and both TNF-α and fatty acids probably due to an uptake of D-FA followed by cell inflammation (Fig. 9c). Those findings may suggest a different way of interaction of D-FA with the cell membrane depending on the length of their hydrocarbon chains, which may affect the transport of FA into the cell interior.

## Conclusions

Three Raman microscopic methods (i.e., spontaneous Raman, CARS, SRS), together with fluorescence and AFM imaging, were applied to study the uptake of saturated FA by EC in vitro*,* and the effect of inflammation on the ability of EC to accumulate FA and form LD. Two deuterium-labeled saturated FA with different chain lengths, myristic and palmitic acids, were selected and used at two concentrations, 50 µM (low) and 400 µM (high). To follow the accumulation of FA in HMEC-1, D_31_-PA and D_27_-MA were used as sensitive Raman probes to distinguish endogenous FA from those captured by cells. Raman spectroscopy provided detailed spatial and chemical information on the distribution and content of lipids in EC. Fluorescence imaging was used to assess EC inflammation caused by TNF-α and/or by FA uptake. AFM gave an insight into the morphology of the EC.

Each spectroscopic technique viewed the deuterated acid capture process from a different perspective. Imaging with spontaneous Raman and SRS allowed for the separate analysis of signals from endogenous and exogenous FA, on a single cell or whole population scale, respectively. CARS, similarly to fluorescence microscopy, provided information on global lipid content, either by direct measurement or by means of a dye, respectively. Therefore, the use of the four spectroscopic techniques showed how complementary each other was, and that each of them tracked the capture phenomenon, but recognized the process from a different perspective.

Tracking the uptake of D-FA with the use of Raman technique revealed that this process depends on the type of applied FA and their concentration. D_27_-MA used in low concentration is internalized more effectively than D_31_-PA, the reversed results for D_27_-MA and D_31_-PA uptake were obtained when high concentration was used. Inflammation evoked by TNF-α treatment upregulated the uptake of D_27_-MA in low concentration, which was not observed for the incubation of inflamed cells with higher concentrations of D-FA. The reduced amount of exogenous FA in inflamed cells may indicate a slight downregulation of the FA uptake and the lipid accumulation capacity of EC that occurs as a result of inflammation. During inflammation, the impaired ability of the endothelium to respond to environmental changes, e.g., ineffectiveness in buffering and transporting lipids from plasma, may contribute to the disturbance of the homeostasis of the whole organism and lead to, e.g. the development of cardiovascular diseases. The differences in the uptake of FA (here investigated by using isotope labeling) proved the complexity of this process. The incubation with D-FA did not increase the inflammation of EC, nor did it affect the overall content of lipids, as provided by fluorescence microscopy. It is worth investigating further whether the differences observed with TNF-α are associated with changes in FA release and/or their utilization by the cells.

Analysis of the morphology of the EC incubated with D_27_-MA indicated their characteristic spindle shape and higher height compared to the EC treated with D_31_-PA, which in turn were more oval and had a rougher cell membrane. These findings could suggest a different way of FA interaction with the cell depending on the length of their hydrocarbon chains, which could affect the transport of FA across the cell membrane.

To conclude, a detailed analysis of inflamed EC incubated with D-FA was performed. It allowed verifying the hypothesis of whether EC capture more FA in the state of inflammation. This seems to be the case for the inflammation of EC incubated with D_27_-MA at 50 µM, while for D_31_-PA it was rather at a higher concentration (400 µM).

## Data Availability

The datasets generated during and/or analysed during the current study are available from the corresponding author on reasonable request.

## Abbreviations

AFM: Atomic force microscopy
CARS: Coherent anti-Stokes Raman scattering
D-FA: Deuterated fatty acids
D_27_-MA: Myristic-D_27_ acid
D_31_-PA: Palmitic-D_31_ acid
EC: Endothelial cells
ER: Endoplasmic reticulum
FA: Fatty acids
HMEC-1: Human dermal microvascular endothelial cells
ICAM-1: Intercellular adhesion molecule 1
KMCA: *k-*Means cluster analysis
LD: Lipid droplets
MA: Myristic acid
PA: Palmitic acid
SRS: Stimulated Raman scattering
TNF-α: Tumor necrosis factor-alpha
